# Wall shear stress and relative residence time as potential risk factors for abdominal aortic aneurysms in males: a 4D flow cardiovascular magnetic resonance case–control study

**DOI:** 10.1186/s12968-022-00848-2

**Published:** 2022-03-18

**Authors:** Chiara Trenti, Magnus Ziegler, Niclas Bjarnegård, Tino Ebbers, Marcus Lindenberger, Petter Dyverfeldt

**Affiliations:** 1 Unit of Cardiovascular Sciences, Department of Health, Medicine and Caring Sciences, Linköping University, Linköping, Sweden; 2 Center for Medical Image Science and Visualization (CMIV), Linköping University, Linköping, Sweden; 3 Department of Cardiology in Linköping, and Department of Health, Medicine and Caring Sciences, Linköping University, Linköping, Sweden

**Keywords:** Abdominal aortic aneurysm, 4D flow, Wall shear stress, Oscillatory shear index, Relative residence time

## Abstract

**Background:**

Abdominal aortic aneurysms (AAA) can lead to catastrophic events such as dissection or rupture, and are an expression of general aortic disease. Low wall shear stress (WSS), high oscillatory shear index (OSI), and high relative residence time (RRT) have been correlated against increased uptake of inflammatory markers in the vessel wall and may improve risk stratification of AAA. We sought to obtain a comprehensive view of WSS, OSI, and RRT in the whole aorta for patients with AAA and age-matched elderly controls and young normal controls.

**Methods:**

4D Flow cardiovascular magnetic resonance images of the whole aorta were acquired in 18 AAA patients (70.8 ± 3.4 years), 22 age-matched controls (71.4 ± 3.4 years), and 23 young subjects (23.3 ± 3.1 years), all males. Three-dimensional segmentations of the whole aorta were created for all timeframes using a semi-automatic approach. The aorta was divided into five segments: ascending aorta, arch, descending aorta, suprarenal and infrarenal abdominal aorta. For each segment, average values of peak WSS, OSI, and RRT were computed. Student’s t-tests were used to compare values between the three cohorts (AAA patients vs elderly controls, and elderly controls vs young controls) where the data were normally distributed, and the non-parametric Wilcoxon rank sum tests were used otherwise.

**Results:**

AAA patients had lower peak WSS in the descending aorta as well as in the abdominal aorta compared to elderly controls (*p* ≤ 0.001), similar OSI, but higher RRT in the descending and abdominal aorta (*p* ≤ 0.001). Elderly controls had lower peak WSS compared to young controls throughout the aorta (*p* < 0.001), higher OSI in all segments except for the infrarenal aorta (p < 0.001), and higher RRT throughout the aorta, except the infrarenal aorta (*p* < 0.001).

**Conclusions:**

This study provides novel insights into WSS, OSI, and RRT in patients with AAA in relation to normal ageing, highlighting how AAA patients have markedly abnormal hemodynamic stresses not only in the infrarenal, but in the entire aorta. Moreover, we identified RRT as a marker for abnormal AAA hemodynamics. Further investigations are needed to explore if RRT or other measures of hemodynamics stresses best predict AAA growth and/or rupture.

**Supplementary Information:**

The online version contains supplementary material available at 10.1186/s12968-022-00848-2.

## Background

Abdominal aortic aneurysm (AAA) is characterized by a progressive dilation of the abdominal aorta, primarily the infrarenal aorta. The largest risk associated with AAA is rupture, a catastrophic event associated with a mortality of 50–80% [1, 2]. Recent studies indicate that the prevalence of AAA in 65–70-year-old males is approximately 1.5–3% in Europe [2–4]. Current European guidelines suggest surveillance imaging of the aortic diameter and elective surgery when the diameter reaches 5.5 cm [5]. In addition to the rupture risk, AAA significantly increases the risk of other cardiovascular events, advancing the hypothesis that AAA is an expression of a general cardiovascular disease [6]. For example, the co-prevalence of AAA and thoracic aortic aneurysm is 10–30% [7–9]. Further, recent studies associated AAA with increased left-ventricular workload and faster ageing of the central arteries [10, 11].

Aortic aneurysms lead to altered aorta hemodynamics. Of particular interest is wall shear stress (WSS), which represents the frictional force of blood on the vessel wall. WSS and oscillatory shear index (OSI) have previously been correlated with upregulation of inflammatory markers, and an increase in oxidative activities of endothelial cells in vitro [12]. Additionally, elevated OSI in a model of human abdominal aorta have been associated with intima thickness of the atherosclerotic wall in 15 post mortem aortas [13]. Himburg et al. [14] investigated the relation between endothelial permeability and shear stress in the porcine abdominal aorta, and identified an increased macromolecular uptake in regions of low and oscillatory shear stress, suggesting that these two different shear aspects could be combined into a new marker, relative residence time (RRT). Elevated RRT has been found in the inner wall of the aortic arch of hypertensive rats, corresponding to regions of extensive elastin degradation of the tunica media near the inner wall of the aortic arch [15]. Further, disturbed and stagnant flow, expressed by elevated RRT, was found close to atherosclerotic lesions in a model of aortic regurgitation of murine aortas [16].

Previous studies of WSS and OSI in patients with AAA are limited [17]. One study based on patient-specific models found that low WSS appears to predominate at sites of AAA rupture, but this finding was based on computational fluid dynamics simulations of only six patients with AAA [18]. WSS can also be assessed with three-dimensional (3D), time-resolved phase-contrast cardiovascular magnetic resonance (CMR) imaging with three-directional velocity encoding (4D flow CMR), that allows for 3D blood flow quantification in the vasculature throughout the cardiac cycle [19]. While 4D flow CMR has widely been used to investigate altered WSS in the diseased thoracic aorta [20–22], previous studies of WSS and OSI in patients with AAA are limited. Takehara et al. [23] found reduced peak WSS values and elevated OSI in AAA when compared to non-dilated proximal aortic segments in a cohort of 18 AAA patients. However, no control group was studied. Sughimoto et al. [24] derived similar findings in 8 young healthy subjects, suggesting that elevated OSI in the infrarenal aorta may be one of the factors that lead to morphological changes over time. Unfortunately, no AAA patients were included in this study.

In spite of the previous efforts to study hemodynamics in AAA, studies comparing WSS and OSI in patients with AAA against controls are lacking, while the potential of RRT as a marker for altered aneurysmal hemodynamics has been explored only in animal studies so far. Therefore, the aim of this study was to assess inter-cohort differences in WSS, OSI, and RRT in a population of AAA male patients, age-matched elderly non-dilated male controls and young healthy male controls. Comparisons were made to assess the effect of disease (AAA patients vs elderly controls) and normal ageing (young controls vs elderly controls). Further, in order to provide more context for the interpretation of WSS and OSI in the abdominal aorta and to explore the association between AAA and hemodynamics in thoracic aorta, we set out to explore WSS, OSI, and RRT in the entire aorta.

## Methods

### Data acquisition and processing

18 AAA male patients (66–76 years) and 22 age- and sex-matched controls were recruited from an AAA screening program at Linköping University Hospital. AAA patients presented with maximum abdominal aortic diameters ≥ 3.5 cm, while matched controls had no focal dilation and diameters < 2.5 cm in the abdominal aorta on ultrasound. Additionally, 23 sex-matched young (18–30 years) subjects were enrolled. Inclusion criteria included sinus rhythm, and no contraindications for CMR. Characteristics for the three study cohorts are summarized in Table 1. The study was approved by the regional ethical review board and all subjects gave written informed consent.

**Table 1:** Overview of cohorts’ characteristics, presented as mean ± (standard deviation)

	AAA	Elderly controls	Young controls	AAA vs elderly controls (*p*)	Elderly controls vs young controls (*p*)
N	18	22	23		
Age	70.8 ± (3.4)	71.4 ± (3.4)	23.3 ± (3.1)	0.713	< 0.001*
Height [cm]	176.6 ± (6.7)	177.6 ± (6.5)	182.5 ± (6.6)	0.783	0.011*
Weight [kg]	86.6 ± (10.3)	81.9 ± (8.7)	78.3 ± (10.3)	0.153	0.189
BSA [m^2^]	2.1 ± (0.1)	2.0 ± (0.1)	2.0 ± (0.2)	0.255	0.665
Heart rate [bpm]	63.2 ± (9.8)	64.8 ± (12.1)	62.6 ± (10.0)	0.627	0.473
Stroke volume [ml/min]	64.4 ± (12.5)	65.0 ± (17.4)	79.2 ± (14.5)	0.903	0.005*
Systolic blood pressure[mmHg]	133.4 ± (13.7)	122.9 ± (14.9)	110 ± (7.0)	0.031	< 0.001*
Diastolic blood pressure[mmHg]	74.3 ± (7.3)	72.3 ± (7.9)	58.5 ± (3.8)	0.44	< 0.001*
Smoking				< 0.001**	< 0.001**
Current	2 (11.1%)	0 (0%)	0 (0%)		
Quit	16 (88.2%)	11 (50%)	1 (4.3%)		
Never	0 (0%)	11 (50%)	22 (95.6%)		
Diabetes mellitus				1	0.489
Yes	1 (5.5%)	1 (4.5%)	0 (0%)		
No	17 (94.5%)	21 (95.5%)	23 (100%)		
Hypertension				0.125	< 0.001**
Yes	12 (66.6%)	9 (40.9%)	0 (0%)		
No	6 (33.4%)	13 (59.1%)	23 (100%)		
Previous cardiac disease				1	0.0491
Yes	4 (22.2%)	4 (18.2%)	0 (0%)		
No	14 (77.8%)	18 (81.8%)	23 (100%)		
Previous lung disease				0.642	0.233
Yes	3 (16.6%)	2 (9.1%)	0 (0%)		
No	15 (83.4%)	20 (90.0%)	23 (100%)		
Previous cerebrovascular disease				1	0.489
Yes	1 (5.5%)	1 (4.5%)	0 (0%)		
No	17 (94.5%)	21 (95.5%)	23 (100%)		
Previous renal disease				1	0.489
Yes	0 (0%)	1 (4.5%)	0 (0%)		
No	18 (100%)	21 (95.5%)	23 (100%)		

Systolic and diastolic blood pressure reported as average of two measures from carotid ultrasound (PWVcr) performed before CMR examination

*AAA* abdominal aortic aneurysm, *BSA* body surface area

^*^Indicates statistical difference (*p* < 0.025) with Student t-test; **indicates statistical difference (*p* < 0.025) with Fisher exact test for categorical data

All subjects underwent a CMR exam at a 3T CMR system (Ingenia, Philips Healthcare, Best, The Netherlands) equipped with a 32-channel torso coil with 60 cm coverage. Balanced steady-state free precession (bSSFP) images were acquired and used to assess aneurysm and thrombus morphology. Scan parameters included: axial slab covering the abdominal aorta, flip angle 60°, echo time 1.5 ms, repetition time 3.1 ms, and spatial resolution 1.4 × 1.4 × 5 mm. AAA morphology was visually determined by one observer (Table 2). Contrast-enhanced CMR angiography (CE-CMRA) volumes were acquired following administration of a gadolinium contrast agent (Magnevist, Bayer Healthcare, Berlin, Germany) and used to determine relevant aortic landmarks. Scan parameters included: a sagittal-oblique slab set to cover the whole aorta from left ventricular outflow tract to iliac bifurcation, flip angle 30°, echo time 2 ms, repetition time 5.8 ms, parallel imaging (SENSE) factor 4, and spatial resolution 0.6 × 0.6 × 1.2 mm. 4D flow CMR data were acquired with a free-breathing, respiratory navigator gated, retrospectively cardiac-gated sequence immediately after the CE-CMRA. The 4D flow sequence used a center-out acquisition strategy to acquire the central parts of k-space while there was still contrast agent in the blood. Scan parameters included: a sagittal-oblique slab with 3D field of view (FOV) = 480 – 560 × 480 – 560 × 71 – 117 mm^3^ and matrix size = 192 – 224 × 192 – 224 × 28 – 46 adjusted depending on each subject’s anatomy to cover the whole aorta from the aortic valve to the iliac bifurcation, velocity encoding range (VENC) 100–200 cm/s, flip angle 15°, echo time = 2.5–3.1 ms, repetition time = 4.4–5.0 ms, k-space segmentation factor 2, effectively acquired temporal resolution 35–40 ms, and acquired spatial resolution 2.5 × 2.5 × 2.5 mm^3^. SENSE parallel imaging was used, with a total acceleration factor of 4–4.8. Data were reconstructed to 40 timeframes. Total scan time including respiratory navigator gating was 10–15 min. 4D flow datasets were corrected for concomitant gradient field effects on the scanner, while phase-wraps and background phase-offset errors were corrected offline [25, 26]. Background phase-offset errors were corrected using a weighted 4th order fit to static tissue [26].

**Table 2:** Overview of AAA patients characteristics

	AAA diameter (echo) [mm]^a^	Type	Presence of ILT
AAA01	45	Saccular	Medium
AAA02	41	Fusiform	Medium
AAA03	39	Fusiform	Absent
AAA04	44	Fusiform	Medium
AAA05	52	Saccular	Small
AAA06	50	Fusiform	Absent
AAA07	42	Fusiform	Large
AAA08	44	Fusiform	Absent
AAA09	40	Fusiform	Not visible
AAA10	52	Fusiform	Large
AAA11	39	Fusiform	Small
AAA12	39	Saccular	Small
AAA13	54	Fusiform	Large
AAA14	49	Fusiform	Large
AAA15	39	Fusiform	Not visible
AAA16	43	Fusiform	Small
AAA17	48	Fusiform	Medium
AAA18	41	Saccular	Medium

*ILT* intraluminal thrombus

^a^Maximum abdominal aortic diameter measured during echocardiography examination

### Time-resolved segmentation

Phase-contrast CMR (PC-CMR) images were computed from the magnitude and velocity images at peak systole, defined as the timeframe with maximum mean velocity in the whole aorta. 3D segmentation was performed using ITK-snap with a semi-automatic region growing algorithm combined with manual adjustments [27]. The 3D segmentation was registered to every other timeframe in the 4D flow CMR data by using a non-rigid registration method based on the Morphon algorithm. The Morphon algorithm uses diffeomorphic field accumulation together with fluid and elastic regularization of the displacement fields in order to generate physically plausible deformations [28]. Consequently, isosurfaces at each timeframe were generated and registered to the peak systolic isosurface with a non-rigid variant of the iterative closest point algorithm [29]. In this way, the same node on the peak systolic isosurface could be tracked on isosurfaces at each timeframe, allowing for computation of time dependent parameters.

### WSS analysis

Instantaneous WSS vectors were computed with a previously described method [30].The WSS computation was conducted using three points on the inward normal vector and a length of the vector itself of 12 mm, 10 mm and 9 mm for AAA patients, elderly controls and young controls, respectively. This cohort-specific inward vector took into account the differences in aortic lumen size between the cohorts, and fall within the optimum range as defined by Potters et al. [30]. A Carreu-Yasuda model was used for viscosity with viscosity at infinite shear rate assumed to be 0.0035 [Pa s]. Based on the instantaneous WSS vectors, the time-averaged wall shear stress (TAWSS) [Pa] through cardiac cycle were computed as:

$$TAWSS= \frac{1}{T}{\int }_{0}^{T}\left|WSS dt\right|.$$

Further, the oscillatory shear index (OSI) [−] was computed as

$$OSI= 0.5 * \left(1 - \frac{\left|\underset{0}{\overset{T}{\int }}WSSdt\right|}{{\int }_{0}^{T}\left|WSS\right|dt}\right).$$

Finally, the relative residence time (RRT)[−], which estimates the relative duration that blood resides close to the wall through a combination of TAWSS and OSI, was computed as in [14]

$$RRT \sim \frac{1}{\left(1-2*OSI\right)*TAWSS}.$$

**Fig. 1:**
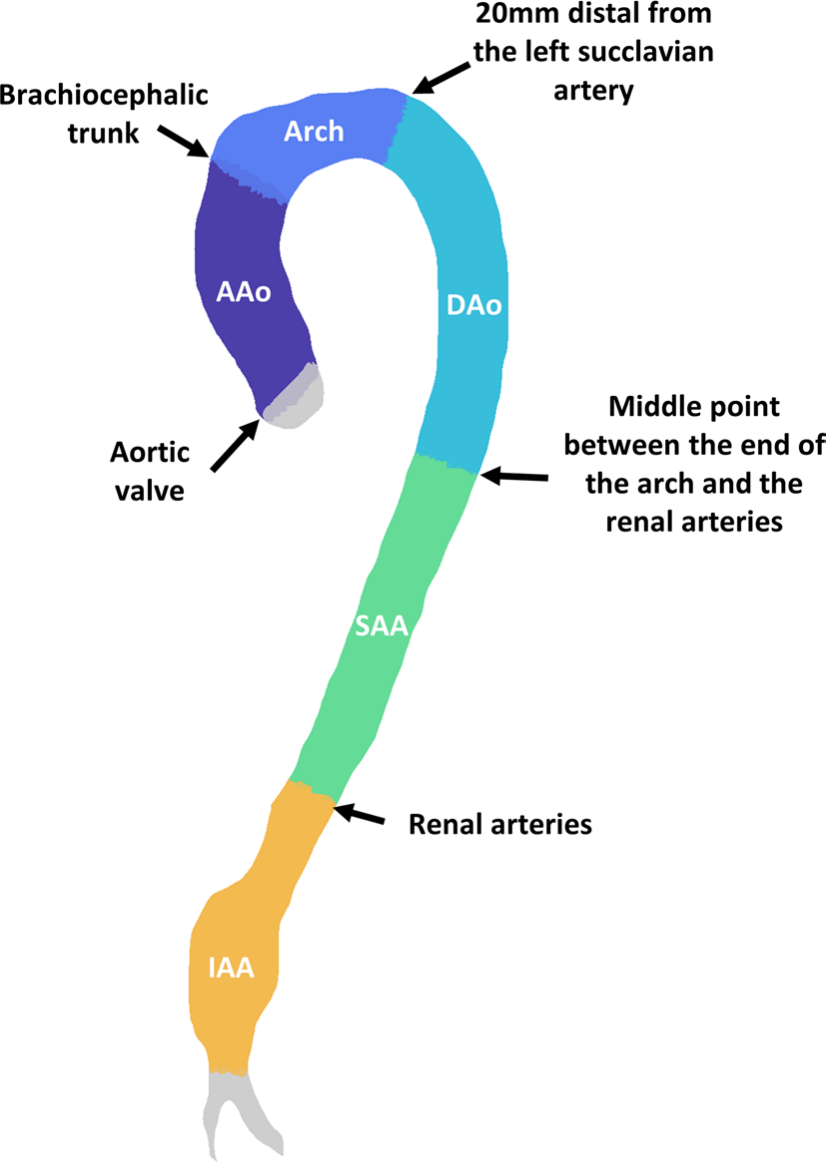
Landmarks identified on contrast-enhanced cardiovascular magnetic resonance angiogram (CE-CMRA) aortic images used to divide the aorta in 5 different segments: ascending aorta (AAo)—from the valve to brachiocephalic trunk, arch—from brachiocephalic trunk to the point 20 mm distal from the left subclavian artery, descending aorta (DAo)—from the distal end of the arch to midway between the end of the arch and the renal arteries, suprarenal abdominal aorta (SAA)—from the distal end of DAo to renal arteries, infrarenal abdominal aorta (IAA)—from the renal arteries to the iliac bifurcation

The aorta was divided into five segments and WSS parameters were computed in each segment. First, five landmarks were determined by visual inspection of one expert observer on CE-CMRA images: aortic valve, brachiocephalic trunk and left subclavian insertion on the arch, renal arteries and iliac bifurcation. These annotations were registered to the 4D flow images, projected on the centreline of the aorta, and subsequently used to define five segments (Fig. 1): ascending aorta (AAo)—from the valve to brachiocephalic trunk; arch—from brachiocephalic trunk to the point 20 mm distal from the left subclavian artery; descending aorta (DAo)—from the distal end of the arch to midway between the end of arch and the renal arteries; suprarenal abdominal aorta—from the distal end of DAo to renal arteries; infrarenal abdominal aorta—from the renal arteries to the iliac bifurcation. The time frame with peak flow rate (TF_peak_) were identified for each segment [31]. Average velocity and average WSS at TF_peak_ (peak velocity and peak WSS, respectively) were computed in each segment. Additionally, mean TAWSS, OSI, and RRT were computed for each segment. Finally, the maximum diameter normalized by body surface area (BSA), was obtained for each segment based on the cross-sectional area.

Further, a local analysis was performed to identify the extent to which the infrarenal abdominal aorta was exposed to abnormal WSS. Abnormally low peak WSS was defined as WSS values more than two standard deviations lower than the mean WSS in the elderly cohort. Similarly, abnormally high OSI and RRT were defined as values more than two standard deviations higher than the mean values in the elderly cohort. The area affected by abnormal values was computed and compared to the elderly cohort.

Finally, to assess the impact of the intraluminal thrombus on WSS parameters, the intraluminal thrombus was segmented on the bSSFP images by one operator on all 13 AAA patients in which the intraluminal thrombus was visually identifiable. The bSSFP images were registered to the 4D Flow images to identify the interface between the lumen and intraluminal thrombus. Average values of peak WSS, TAWSS, OSI and RRT acting on the entire infrarenal abdominal aorta and the thrombus-free wall in the infrarenal abdominal aorta were compared.

### Statistical analysis

The Shapiro–Wilk test was used to test the normality of the distribution of each parameter in each segment. Results were expressed by means of mean ± (standard deviation) when normally distributed and median [interquartile range] otherwise. Moreover, when normally distributed, a two-tailed unpaired Student’s t-test was used to compare AAA vs elderly controls, and young controls vs elderly controls. Bonferroni correction for multiple comparison was applied and a p value < 0.025 (0.05/2) was considered significant. If not normally distributed, a non-parametric Wilcoxon rank sum test was used. Two-tailed unpaired student’s t-test was also used to compare values between the infrarenal abdominal aorta and suprarenal abdominal aorta, when data were both normally distributed, and a non-parametric Wilcoxon rank sum test was used when data were not normally distributed.

## Results

**Fig. 2:**
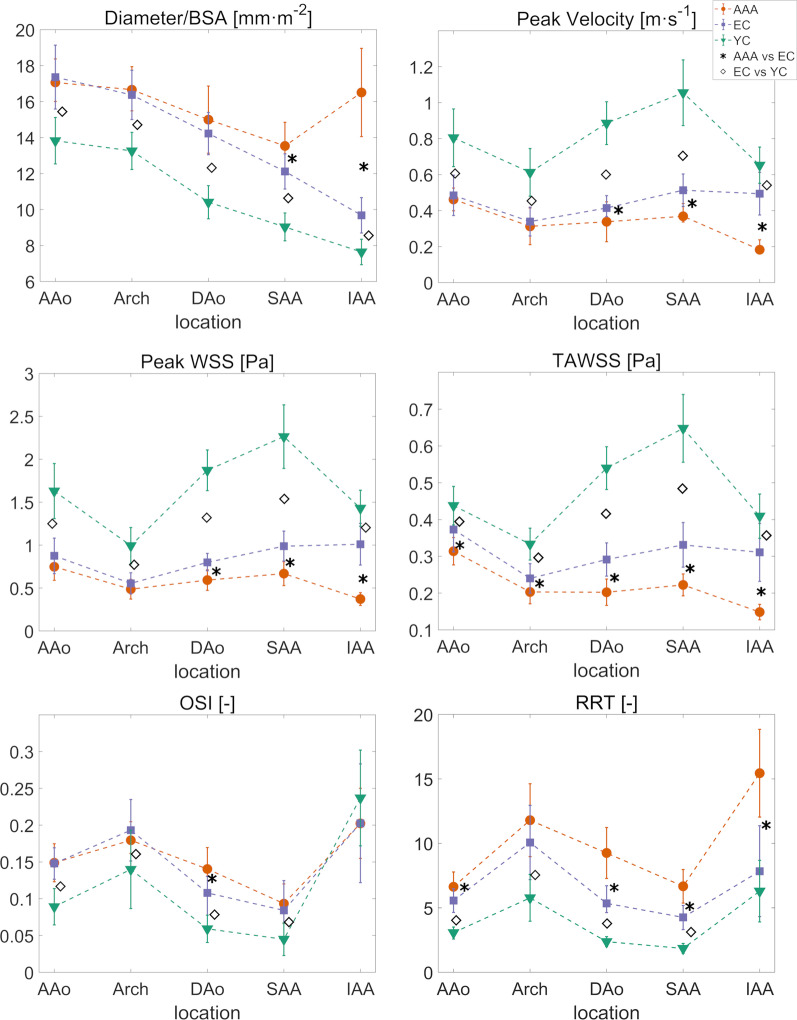
For each segment, average values of diameter normalized by body surface area (BSA), velocity, wall shear stress (WSS), time averaged wall shear stress (TAWSS), oscillatory shear index (OSI), and relative residence time (RRT) are represented for the three cohorts: AAA (red line with circular markers), elderly controls (blue line with squared markers), young controls (green line with triangles). Stars indicate significant difference between AAA and elderly controls (*p* < 0.025), diamonds between elderly and young controls (*p* < 0.025). Symbols and bars indicate mean and standard deviation in the cohort when the population is normally distributed, median and interquartile range values when not. *EC* elderly controls, *YC* young controls, *SAA* suprarenal abdominal aorta, *IAA* infrarenal abdominal aorta, *DAo*, descending aorta

For each segment, average values of diameter normalized by BSA, peak velocity, peak WSS, TAWSS, OSI, and RRT are reported in Table 3 and Fig. 2 for the three cohorts.

**Table 3:** Results for the whole aorta in the three cohorts

	AAA (N = 18)	Elderly controls (N = 22)	Young controls (N = 23)	AAA vs elderly controls (*p*)	Elderly controls vs young controls (*p*)
Diameter/BSA [mm m^−2^]					
AAo	17.07 [2.36]	17.36 ± (1.77)	13.82 ± (1.29)	0.9026	< 0.001*
Arch	16.66 [2.46]	16.37 ± (1.37)	13.26 ± (1.03)	0.4882	< 0.001*
DAo	15 ± (1.87)	14.22 ± (1.18)	10.41 ± (0.92)	0.1192	< 0.001*
SAA	13.53 ± (1.32)	12.12 ± (0.98)	9.03 ± (0.77)	< 0.001*	< 0.001*
IAA	16.51 ± (2.45)	9.68 ± (0.98)	7.64 ± (0.71)	< 0.001*	< 0.001*
Peak velocity [m s^−1^]					
AAo	0.46 [0.13]	0.48 ± (0.11)	0.8 ± (0.16)	0.577	< 0.001*
Arch	0.31 ± ( 0.1)	0.34 ± (0.08)	0.61 ± (0.13)	0.363	< 0.001*
DAo	0.34 ± (0.11)	0.41 [0.12]	0.89 ± (0.12)	0.005*	< 0.001*
SAA	0.37 [ 0.1]	0.51 ± (0.09)	1.06 ± (0.18)	< 0.001*	< 0.001*
IAA	0.18 [0.07]	0.49 ± (0.12)	0.65 ± ( 0.1)	< 0.001*	< 0.001*
Peak WSS [Pa]					
AAo	0.75 ± (0.16)	0.88 ± (0.21)	1.63 ± (0.32)	0.038	< 0.001*
Arch	0.49 ± (0.12)	0.55 ± (0.12)	0.99 ± (0.21)	0.083	< 0.001*
DAo	0.59 ± (0.12)	0.8 [ 0.2]	1.87 ± (0.24)	< 0.001*	< 0.001*
SAA	0.67 ± (0.14)	0.99 ± (0.17)	2.27 ± (0.37)	< 0.001*	< 0.001*
IAA	0.37 ± (0.07)	1.01 ± (0.24)	1.43 ± (0.21)	< 0.001*	< 0.001*
TAWSS [Pa]					
AAo	0.31 ± (0.04)	0.37 ± (0.06)	0.44 ± (0.05)	< 0.001*	< 0.001*
Arch	0.2 ± (0.03)	0.24 ± (0.04)	0.33 ± (0.04)	0.003*	< 0.001*
DAo	0.2 ± (0.04)	0.29 ± (0.05)	0.54 ± (0.06)	< 0.001*	< 0.001*
SAA	0.22 ± (0.03)	0.33 ± (0.06)	0.65 ± (0.09)	< 0.001*	< 0.001*
IAA	0.15 ± (0.02)	0.31 ± (0.08)	0.41 ± (0.06)	< 0.001*	< 0.001*
OSI [−]					
AAo	0.15 ± (0.03)	0.15 ± (0.02)	0.09 ± (0.02)	0.900	< 0.001*
Arch	0.18 [0.04]	0.19 ± (0.04)	0.14 ± (0.05)	0.775	< 0.001*
DAo	0.14 ± (0.03)	0.11 [0.05]	0.06 ± (0.02)	0.006*	< 0.001*
SAA	0.09 ± (0.03)	0.08 ± (0.04)	0.04 ± (0.02)	0.392	< 0.001*
IAA	0.2 ± (0.05)	0.2 ± (0.08)	0.24 ± (0.07)	0.946	0.122
RRT [−]					
AAo	6.63 ± (1.15)	5.56 ± (0.92)	3.07 [0.93]	0.002*	< 0.001*
Arch	11.79 ± (2.83)	10.07 ± (2.88)	5.77 ± (1.81)	0.065	< 0.001*
DAo	9.26 ± (1.97)	5.34 [2.09]	2.37 ± (0.38)	< 0.001*	< 0.001*
SAA	6.67 ± ( 1.3)	4.26 [1.86]	1.85 ± (0.38)	< 0.001*	< 0.001*
IAA	15.44 ± ( 3.4)	7.84 ± (3.53)	6.29 ± (2.39)	< 0.001*	0.091

For each segment, maximum diameter normalized by BSA and average values of peak velocity, peak WSS, TAWSS, OSI and RRT computed from 4D flow CMR are reported as mean ± (standard deviation), when the values are not normally distributed, or median [interquartile range] when not

*AAo* ascending aorta, *DAo* descending aorta, *IAA* infrarenal abdominal aorta, *SAA* suprarenal abdominal aorta

*Indicates *p* ≤ 0.025

### AAA patients vs elderly controls

AAA patients and elderly presented with similar diameters, peak velocities, and peak WSS in the AAo and the arch, whereas AAA had higher diameters in the abdominal aorta (*p* ≤ 0.001 in suprarenal abdominal aorta and < 0.001 in IAA), and lower velocities (*p* = 0.0053 in DAo, *p* ≤ 0.001 in suprarenal abdominal aorta and < 0.001 in infrarenal abdominal aorta), as well as lower peak WSS (*p* < 0.001 in DAo, suprarenal abdominal aorta and infrarenal abdominal aorta) in the descending and abdominal aorta. TAWSS were lower in AAA in each segment (*p* ≤ 0.001 in AAo, *p* = 0.0031 in arch and < 0.001 in DAo, suprarenal abdominal aorta and infrarenal abdominal aorta). AAA patients and elderly controls had similar OSI in the whole aorta, except in the DAo (*p* = 0.0047). RRT is similar between the two groups in the arch, whereas AAA patients have higher RRT (*p* = 0.0023 in AAo and *p* < 0.001 in DAo, suprarenal abdominal aorta and infrarenal abdominal aorta) in the rest of the aorta (Table 3 first and second column, and Fig. 2 red and blue graphs).

While in elderly controls peak WSS and TAWSS values are constant in whole descending aorta, peak WSS and TAWSS decrease from suprarenal abdominal aorta to infrarenal abdominal aorta in AAA patients (*p* < 0.001). OSI and RRT increase from suprarenal abdominal aorta to infrarenal abdominal aorta in AAA patients (*p* < 0.001), as well as in elderly controls (*p* < 0.001).

### Elderly controls vs young controls

Elderly controls had larger diameters, lower peak velocities, lower peak WSS, and lower TAWSS values than young subjects in each segment (*p* ≤ 0.001). Moreover, elderly controls had higher OSI in all segments except in the infrarenal abdominal aorta (*p* ≤ 0.001). Elderly controls had almost doubled RRT compared to young controls in all segments, except for the infrarenal abdominal aorta (*p* < 0.001) (Table 3 second and third column, and Fig. 2 blue and green graphs).

In elderly controls peak WSS and TAWSS mean values were similar in the suprarenal abdominal aorta and infrarenal abdominal aorta, while in young controls, peak WSS and TAWSS were higher in the suprarenal abdominal aorta when compared to the infrarenal abdominal aorta (*p* < 0.001). In both elderly and young controls, OSI and RRT were higher in the infrarenal abdominal aorta when compared to the suprarenal abdominal aorta (*p* < 0.001).

Blood flow in infrarenal abdominal aorta was visualized with streamlines for one AAA (left), one elderly control (central) and one young control (right) in Additional file 1. In the AAA a vortex is visible during diastole, which is not present in the abdominal aorta of the controls. For the same subjects, 3D WSS maps in the infrarenal abdominal aorta over the cardiac cycle are shown in Additional file 2.

### Local analysis of infrarenal abdominal aorta

**Fig. 3:**
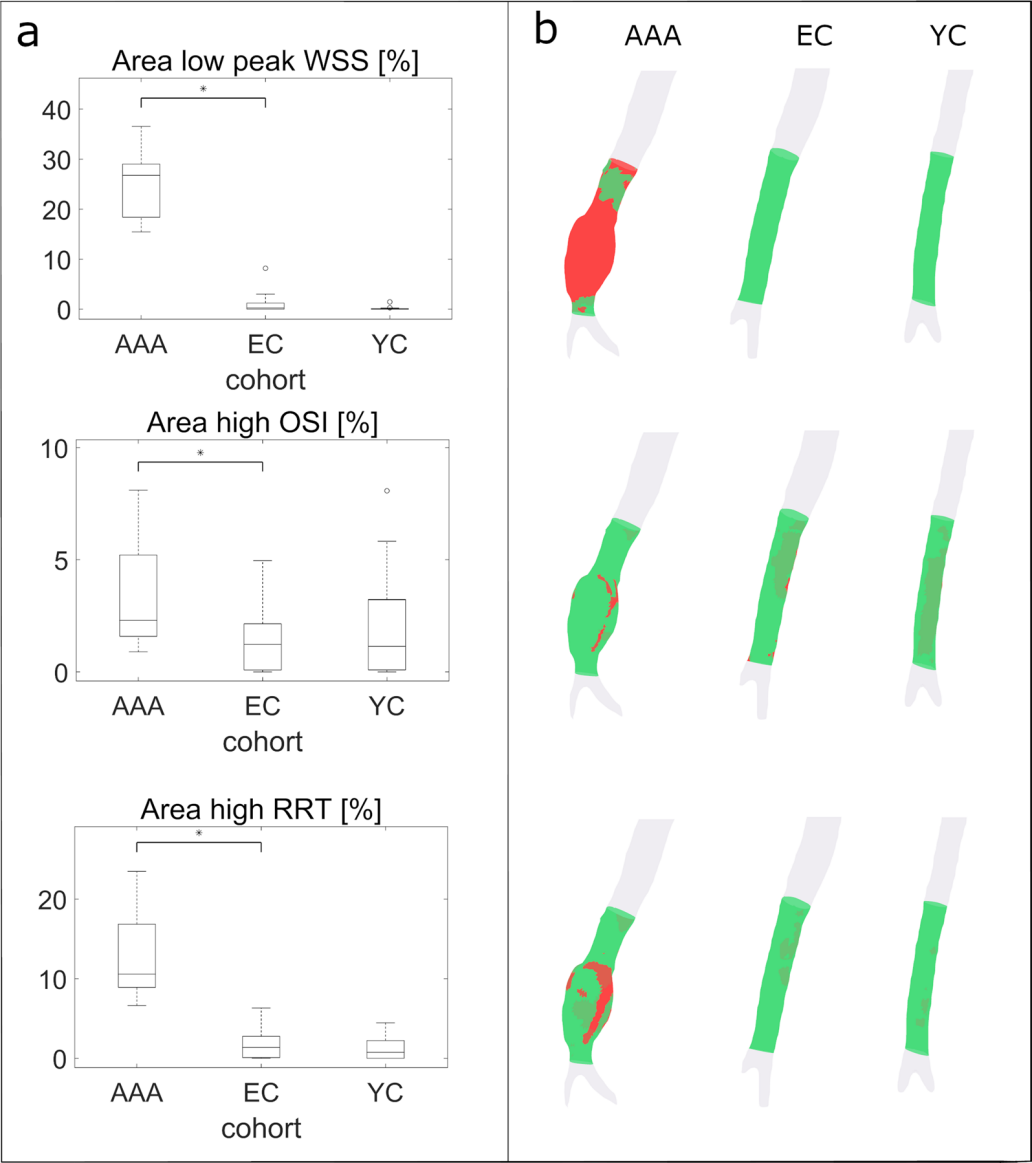
**a** Percentage of area in infrarenal abdominal aorta with abnormally low WSS (top), abnormally elevated OSI (central) and RRT (bottom) in the three cohorts. The thresholds considered to define abnormal values were 0.57 for WSS, 0.36 for OSI and 18.37 for RRT, respectively. **b** Visualization of the area with abnormal values (in red) for one AAA patient, one elderly control (EC) and one young control (YC)

The mean peak WSS value minus 2 standard deviations in the elderly controls was 0.57 Pa, while mean OSI plus 2 standard deviations and mean RRT plus 2 standard deviations were 0.36 and 18.37, respectively. The percentage of infrarenal abdominal aorta vessel wall area exposed by abnormally low peak WSS was 0.25 [1.2] % in elderly controls and 24.7 [5.9] % in AAA (*p* < 0.001). The exposure of abnormally high OSI in the infrarenal abdominal aorta was lower in elderly controls than AAA patients (2.3 [3.6] % vs 1.2 [2.1] %, *p* = 0.0074). The infrarenal abdominal aorta in the AAA patients was more exposed to abnormally high RRT than in the elderly controls (10.6 [7.9] % vs 1.4 [2.7] %, *p* < 0.001), but there was no difference between elderly and young controls. Sites with elevated RRT were located mainly in posterior infrarenal abdominal aorta in young and elderly controls, whereas regions with high RRT are located inside the aneurysm for AAA patients (Fig. 3).

### Intraluminal thrombus in infrarenal abdominal aorta

**Fig. 4:**
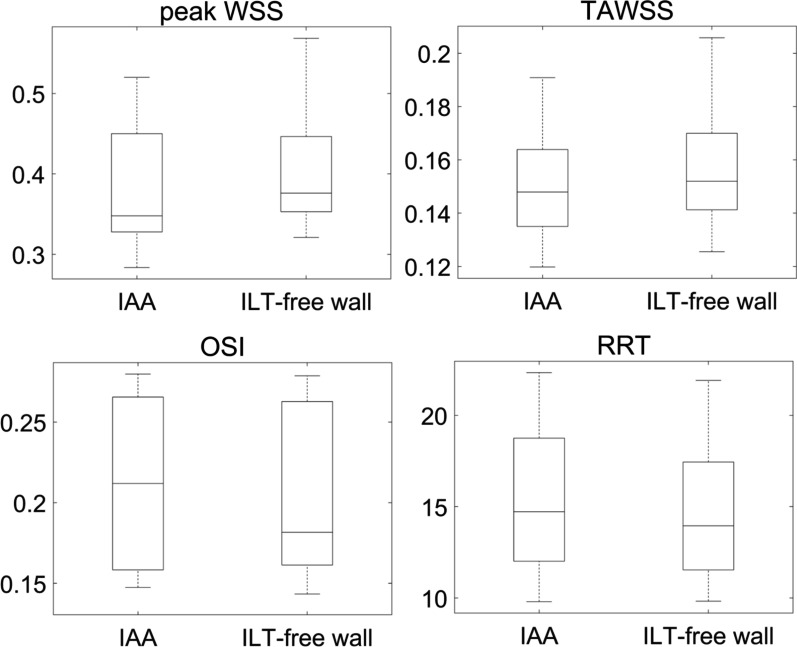
Distributions of average peak WSS (top right), TAWSS (top left), OSI (bottom right), and RRT (bottom left) in the whole infrarenal abdominal aorta and in the intraluminal thrombus (ILT) free wall. Statistical analysis showed no difference between the distributions

Figure 4 shows peak WSS, TAWSS, OSI, and RRT acting on the whole infrarenal abdominal aorta and on the thrombus free vessel wall, respectively, for the 13 AAA patients with an identifiable intraluminal thrombus. For these 13 AAA patients, including or excluding the surface area with intraluminal thrombus did not significantly impact the computation of peak WSS, TAWSS, OSI, or RRT.

## Discussion

This study investigated several expressions of WSS in the aorta of AAA patients and age-matched controls, and provides new insights into AAA in relation to normal ageing by including a population of young subjects. The first main finding of this study is that while age-matched controls experience similar peak WSS and TAWSS in the suprarenal and infrarenal abdominal aorta, the infrarenal abdominal aorta in AAA patients experiences significantly lower WSS than the proximal aorta. This is likely driven by an expansion of the aorta in AAA patients that leads to reduced velocities, as reported in a previous study [23]: holding flow rate constant, mean velocity and thus velocity gradients are reduced in a larger vessel, which leads to a decrease in WSS. Second, our findings reveal that RRT is almost doubled in AAA compared to age-matched controls. Elevated RRT close to the arterial wall could promote the uptake of inflammatory cells and biomarker, contributing to the degeneration the of aortic wall that leads to growth and rupture.

The largest differences between elderly and young cohorts were seen in the DAo and suprarenal abdominal aorta. In young aortas, there is a progressive increase in velocities from the arch till the renal arteries, with a sudden drop in velocity after the renal arteries, which is not explained by the constant decrease in diameter towards the distal portion of the aorta. We speculated that such difference between suprarenal abdominal aorta and infrarenal abdominal aorta could be due partly to high flow to the renal arteries in young controls. Indeed, 25% of cardiac output is headed to the kidney. In the elderly controls, lower in velocities in the proximal segments of the aorta compared to young controls could be explained by an increase in aortic diameter with age, as we have previously reported [31], by decreased in stroke volume (Table 1), but also with an increase in tortuosity with age, which has been reported in previous studies [32, 33]. Further, increased tortuosity might be associated with the age-related increase in OSI and RRT seen in the proximal aortic segments. Age-related effects on the aortic wall are related mainly to degradation of the elastic lamina, which is more pronounced in the proximal aorta [34]. Interestingly, the infrarenal abdominal aorta was the segment were the hemodynamics parameter differ the least between the elderly and young cohorts.

RRT appears to be a more powerful measure of altered AAA hemodynamics than the commonly used OSI, because it was able to distinguish AAA patients from elderly controls. Similar to Takehara et al. [23], we found that OSI is higher in the infrarenal abdominal aorta than the suprarenal abdominal aorta in the AAA patients, which could be explained by the formation non-laminar flow by the presence of the aneurysm. However, more unexpectedly, there was no difference in infrarenal abdominal aorta for OSI between the three cohorts. This is in line with Himburg et al. [14] who suggested that OSI alone is not a suitable index for describing recirculation zones in pulsatile flows, because although OSI can identify regions of flow reversal, it is insensitive to WSS magnitude. We speculate that OSI in the normal-sized infrarenal abdominal aorta is elevated due to reverse flow in early diastole, whereas elevated OSI in the infrarenal abdominal aorta in AAA patients may be caused by recirculation inside the aneurysm during diastole, as shown in the additional movies (Additional files 1, 2). Indeed, the iliac bifurcation creates a reflected pressure-wave that leads to reverse flow during this phase of the cardiac cycle and allows flow from the infrarenal abdominal aorta back into the kidneys [35]. The presence of an AAA impairs the reverse flow, and as a results, it might increase flow stasis, as investigated by Ziegler et al. [36]. Therefore, OSI might better be employed in combination with other measures of WSS. RRT, which takes both TAWSS and OSI into account, was able to differentiate between AAA and controls in our study. We therefore advocate the use of RRT in studies of AAA hemodynamics.

It is widely recognized that AAA disease is associated with cardiovascular events not directly related to the aneurysm, but the extent to which AAA is associated with general aortic disease is less clear [6]. Åström Malm et al. [11] recently reported increased aortic stiffness in males with AAA compared to matched controls based on the carotid-to-femoral pulse wave velocity (PWV) method. Further studies with 4D Flow CMR-based regional PWV estimation may establish whether increased stiffness in AAA patients occurs uniformly throughout the aorta or mainly in the infrarenal abdominal aorta. The present study adds new data on hemodynamic alterations in the aorta in AAA patients. The lower TAWSS seen in the entire aorta and the increased RRT seen in the DAo and suprarenal abdominal aorta may promote aortic wall deterioration in AAA patients.

The role of intraluminal thrombus in AAA is poorly understood. On one hand, intraluminal thrombus seems to play a protective role and limit biomechanical wall stress owing to the increased thickness of the wall [37]. On the other hand, the intraluminal thrombus is a highly proteolytic and oxidative environment and in the aorta, where blood pressure is high, radial convection pushes the intraluminal thrombus proteolytic and oxidative components into the wall, which may contribute to progressive dilatation and risk of rupture [35]. The combination of low velocities and oscillatory flow in the aneurysm lead to flow stagnation that promotes the deployment of fibrinogen, circulating cellular elements, such as leukocytes, platelets, and red blood cells in the intraluminal thrombus. Enzymes and other components released by the cells that aggregate at intraluminal thrombus, are transported outwards to the wall and contribute to extracellular matrix degradation and adventitia immune response [25]. In this light, RRT, is a potential marker to locate sites at risk of for rupture at the intraluminal thrombus surface. A focal analysis of the effect of WSS on the intraluminal thrombus surface was beyond the scope of this study, but could bring new insights to the role of intraluminal thrombus in AAA.

### Limitations and future studies

This study has several limitations. First, the number of AAA patients included is relatively small, so the statistical power of the study is limited. Moreover, inclusion of more patients would have enabled comparisons between different aneurysm shapes, such as fusiform vs saccular. Nevertheless, this number of AAA patients included in this study is larger than in previous studies of hemodynamics in the abdominal aorta. Second, follow-up data on AAA growth and rupture were not available, thus limiting the study to a cross-sectional design. Therefore, it is not possible to determine if alterations in RRT are simply correlated to the presence of AAA or if they could contribute to the development of AAA. Future work includes a longitudinal follow-up which may lead to new insights into the role of the hemodynamics markers studied here. Finally, WSS estimation with 4D flow CMR is limited by spatial resolution effects (30, 38). In particular, the estimated WSS depends on the spatial resolution. Another spatial resolution limitation of the WSS algorithm used here is that at least 8 voxels across the diameter of the targeted vessel are recommended for accurate WSS estimates. This does not hold for the infrarenal abdominal aorta in the young subjects in this study.

## Conclusion

While decreased WSS, and increased OSI and RRT seem to be normal physiological effects of ageing, AAA patients have markedly abnormal hemodynamics stresses not only in the infrarenal abdominal aorta, but in the entire aorta. RRT stands out as a powerful marker of altered AAA hemodynamics and merits increased use. Further investigations are needed to explore if RRT or other measures of hemodynamics stress best predict AAA growth, rupture and intraluminal thrombus deposition.

## Supplementary Information

**Additional file 1**: Flow visualization with streamlines in infrarenal abdominal aorta for one AAA(left), one elderly control (EC, central) and one young control (YC, right). In AAA a vortex is visible during diastole, which is not present in the non-dilated abdominal aorta. Visualization created with Ansys EnSight 2020 R2 (ANSYS Inc.).

**Additional file 2**: Visualization of 3D WSS maps during the cardiac cycle in the infrarenal abdominal aorta for one AAA (left), one elderly control (EC, central) and one young control (YC, right). Visualization created in Matlab (ver. R2020a, the Mathworks, Inc., Natick, Massachusetts, USA).

## Data Availability

The CMR datasets used are available from the Linköping University Hospital for researchers who meet the criteria for access to confidential data. The Institutional Review Board form states that the data obtained from the patients will be stored on secure computers within the Linköping University Hospital.

## Abbreviations

AAo: Ascending aorta
AAA: Abdominal aortic aneurysm
BSA: Body surface area
bSSFP: Balanced steady state free precession
CE-CMRA: Contrast enhanced cardiovascular magnetic resonance angiography
CMR: Cardiovascular magnetic resonance
DAo: Descending aorta
EC: Elderly controls
IAA: Infrarenal abdominal aorta
ILT: Intraluminal thrombus
OSI: Oscillatory shear index
PC: Phase contrast
PWV: Pulse wave velocity
RRT: Relative residence time
TAWSS: Time averaged wall shear stress
TFpeak: Time frame with peak flow rate
WSS: Wall shear stress
YC: Young controls
